# When Tides Run Dry: Exploring an Overlooked Coastal Disturbance and Its Climate Connections

**DOI:** 10.1111/gcb.70976

**Published:** 2026-07-05

**Authors:** Robin P. M. Gauff, Davide De Battisti, Alberto Barausse, Laura Airoldi

**Affiliations:** ^1^ Chioggia Hydrobiological Station “Umberto D’ancona”, Department of Biology University of Padova Chioggia Italy; ^2^ Ifremer Coast La Seyne‐sur‐Mer France; ^3^ NBFC National Biodiversity Future Center Palermo Italy

**Keywords:** desiccation, disturbance, extreme weather, intertidal, sea level anomaly, stress

## Abstract

Weakening polar temperature gradients are associated with increasingly persistent weather patterns, increasing the risk of extreme environmental events. While disturbances, such as marine heat waves, have received growing attention, others, including negative sea level anomalies, remain understudied. Prolonged and extreme low‐water conditions can impose strong physiological stress on coastal organisms and alter community structure, yet such events lack a consistent operational definition, limiting their detection and assessment. Here, we define and quantify extreme negative sea level anomalies, termed Dry Tides, as anomalous (below the 10th percentile of historical records) and prolonged (> 5 days) water level depressions that restrict submersion of intertidal and shallow subtidal organisms. Using a global analysis of tidal gauge records from 25 locations, we show that Dry Tides occur worldwide but are most frequent and intense in microtidal and semi‐enclosed systems. These events typically last one to 2 weeks, with extreme cases up to 97 days and reaching amplitude depressions of up to 37% of the local tidal range. Contrary to expectations based on trends in other climate‐related disturbances, we detect no consistent increase in Dry Tide frequency or intensity over the past three decades, which, together with their dependence on tidal regime, suggests that Dry Tides arise from interacting physical drivers rather than a single cause. We discuss the ecological relevance of Dry Tides in light of existing ecological literature, provide tools for their identification using local tide‐gauge databases, and highlight priorities for integrating physical and ecological observations to better assess their impacts under ongoing climate change.

## Graphical abstract

We define and quantify negative sea‐level anomalies, termed Dry Tides, as anomalous (below the 10th percentile of historical records) and prolonged (> 5 days) water level depressions that restrict submersion of intertidal and shallow subtidal organisms, constituting a major but understudied disturbance. Dry Tides are compound events occurring worldwide.

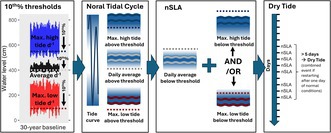

## 1 Introduction

Weather patterns are changing worldwide (Abbass et al. 2022; Pope et al. 2022; Zittis et al. 2022). As temperature gradients towards the poles weaken, jet stream dynamics change, leading to more persistent weather patterns (Francis and Vavrus 2015). As a result, extreme weather events (Francis and Vavrus 2015; Holl 2009; Stott 2016; Tsimplis and Josey 2001) and high‐pressure anomalies (Tsimplis and Josey 2001) increase in intensity and frequency. Some extreme weather events, such as heat waves, have recently attracted significant attention (Hobday et al. 2016; Rousi et al. 2022). However, other potentially detrimental events, such as short‐term sea level changes, remain poorly defined and understudied.

Sea level changes are often associated with extreme weather events like hurricanes (So et al. 2019), heat waves (Mohamed et al. 2019), and other transient weather patterns (Tsimplis and Baker 2000). Storm surges create meter‐high peaks of water levels within days (So et al. 2019), leading to human fatalities and damage to the economy, infrastructures (Davlasheridze et al. 2021; Fang et al. 2014), and ecosystems (Lagomasino et al. 2021). Due to their destructive potential, these short‐term sea level rises have been intensively investigated. In contrast, sea level can also undergo rapid, transient drops. Negative Sea Level Anomalies (nSLAs) have received far less attention, although they can persist for weeks and even months.

These nSLA can cause substantial impacts on intertidal ecosystems. Although direct studies quantifying them remain relatively scarce (e.g., Zamir et al. 2018), mass mortality events have been reported, including the loss of approximately 50% coral cover on a Réunion Island reef (Hoarau et al. 2023). Coral reefs are the ecosystems in which sensitivity to nSLA has been most extensively documented (Ampou et al. 2017; Anthony and Kerswell 2007; Hoarau et al. 2023). However, the potential for severe impacts across a wider range of ecosystems is supported by the extensive literature on the consequences of extraordinary low tides on benthic organisms (Anthony and Kerswell 2007; Fadlallah et al. 1995; Glynn 1968) especially in conjunction with high temperatures, extraordinarily low temperatures or high solar irradiation during midday (Glynn 1968; Hoegh‐Guldberg et al. 2005; Raymundo et al. 2017). The effects of nSLA extend beyond direct biological stress and may also drive broader ecosystem transitions. This is particularly evident in coastal wetlands, where transient sea level depressions open windows of opportunity for plant colonization of mudflats, facilitating their transition into salt marshes or mangroves (Balke et al. 2011; Hu et al. 2015). Such ecosystem shifts are often perceived as positive from a human perspective due to the highly valued services provided by marshes and mangroves (Friess et al. 2020; McKinley et al. 2020; Whitfield 2017). However, mudflat invertebrate communities suffer detrimental impacts from these transitions (Mazik et al. 2010). Thus, nSLA can push communities beyond resistance and resilience thresholds, driving shifts between alternative states, thus qualifying as major, yet overlooked, disturbance in intertidal and shallow subtidal benthic systems (Rykiel Jr 1985; Sutherland 1981).

Beyond their ecological consequences, nSLA also warrant attention because of their broader geographic relevance and potential to affect densely populated coastal regions. In 2023 a strong sea level depression in the Mediterranean Sea was so exceptional as to be covered by information media globally, reporting “*unusually low tides that are making it impossible for gondolas, water taxis and ambulances to navigate some of its [Venice] famous canals*” (The Guardian 2023). In southern France, the sea level was also reported as “*clearly lower than usual*” (Roussel 2023) and the phenomenon seems to have extended to north Africa (Meddi 2023). Negative sea level anomalies are more likely to occur, and be more impactful, in microtidal semi‐enclosed seas, such as the Baltic, Black Sea, and the Mediterranean Sea, where limited tidal range, restricted oceanic exchange, and sensitivity to atmospheric forcing allow sea level anomalies to form easily and persist longer. As coastal ecosystems provide essential ecosystem services to human populations, it is crucial to recognize the relevance of these events.

Negative sea level anomalies have yet to be formally and quantitatively defined, since they encompass a wide range of phenomena, ranging from punctual extreme low tides to month‐long sea level depressions. This lack of a clear definition may partly explain why these events have been largely overlooked, with attention given only to the most extreme cases. Establishing a formal definition would enable their systematic detection worldwide, facilitating the assessment of their impact on benthic systems, and supporting the investigation of potential interactions with other extreme events, such as heatwaves. To address this gap, we provide a quantitative definition of “Dry Tide” disturbances and identify their occurrence using 30 years of historical sea level data from 25 global locations. Additionally, we explore their relationships with climate‐related variables (e.g., atmospheric pressure), examine trends in frequency over time, and provide literature‐based evidence of their ecological relevance.

## 2 Material and Methods

### 2.1 Definition of Dry Tides

Drawing on the hierarchical principles used by Hobday et al. (2016) to define marine heatwaves, we propose the term “Dry Tide”, previously used only informally, as a formal descriptor of *a discrete, prolonged event* (lasting ≥ 5 days) *characterized by anomalously low average sea levels resulting from unusually strong low tides* and/or *weak high tides* (i.e., anomalies in daily tidal peaks). As a consequence, this new definition confines “nSLA” to shorter episodes of similar sea level anomalies.

Specifically, we define:

· *‘anomalous’*: a value that statistically deviates from a local baseline, which is derived from historical observed water level (rather than predicted tidal levels) over an extended period, at least 30 years, as recommended by Hobday et al. (2016). “Dry Tides” and “nSLA” are identified when values fall below the 10th percentile of these historical records. This percentile threshold is not intended to represent a fixed biological tolerance limit; rather, it provides a location‐specific, statistically robust benchmark for identifying unusually low water levels relative to local historical variability, analogous to approaches used for defining other climate‐driven disturbances such as heat waves (Hobday et al. 2016). Because ecological sensitivity to water‐level depression depends strongly on local tidal range, species composition, and habitat structure, absolute thresholds are unlikely to be transferable across systems. The percentile‐based approach therefore facilitates comparability among regions while retaining sensitivity to local conditions. This approach aligns with disturbance ecology frameworks, where departures from background conditions, rather than absolute values, often determine ecological responses, as illustrated by phenomena such as darkening events (Darkwaves; Thoral et al. 2026), dissolved oxygen and acidity extremes (Gruber et al. 2021), and sea surface salinity anomalies (Liu et al. 2023).

To identify a Dry Tide at least two thresholds must be crossed, one by the average daily water level (criterion 1) and one daily extreme, either the maximum or minimum water level, corresponding to peak high or low tide (criterion 2). This approach captures average daily water level depressions resulting from either reduced daily high tides limiting immersion of high‐intertidal organisms or from lowered daily low tides leading to the emersion of organisms not typically exposed to air.

Because sea level baselines are not stationary due to ongoing sea level rise and lunar nodal cycle, these percentile thresholds must be adjusted. As sea level rise varies regionally (Carson et al. 2016; Fasullo and Nerem 2018), we fit a linear regression model integrating a sinusoid lunar nodal cycle of 18.6y of sea level over time for each location. This approach captures local trends and enables computation of a moving 10th percentile threshold.

· *‘prolonged’*: a duration of at least five consecutive days. Days were used rather than tidal cycles because solar irradiation and air temperature, both primarily varying on the diurnal cycle, are the main drivers of stress for intertidal and shallow subtidal organisms, including those not directly emerged during the Dry Tide (Anthony and Kerswell 2007; Glynn 1968; Yamaguchi 1975). The ≥ 5‐day duration threshold follows the hierarchical disturbance framework of Hobday et al. (2016) and is used here as an operational criterion to distinguish short‐lived sea level fluctuations (nSLA) from persistent low‐water disturbances (Dry Tides). While the precise duration required to elicit ecological responses varies among taxa and systems, multiple studies have shown that repeated or prolonged aerial exposure over several consecutive tidal cycles can induce physiological stress, mortality, and shifts in species dominance in intertidal and shallow subtidal communities (Delorme et al. 2021; Guareschi and Wood 2020; Overton et al. 2024). We therefore interpret the five‐day threshold not as a universal biological limit, but as a conservative minimum duration over which low‐water anomalies may plausibly accumulate ecological effects. This threshold is intended to be adaptable and may be refined as additional empirical data become available and our understanding of system‐specific responses improves.

· *‘discrete’*: an event with a clearly defined start and end date, corresponding to the first and last days during which the conditions for a Dry Tide or a nSLA are met. Following Hobday et al. (2016), we treat two Dry Tides events separated by fewer than 2 days as a single continuous event. This merging criterion does not apply to “nSLA” events, as combining short‐duration anomalies could obscure their discrete nature and misrepresent event frequency.

### 2.2 Data Collection

**FIGURE 1:**
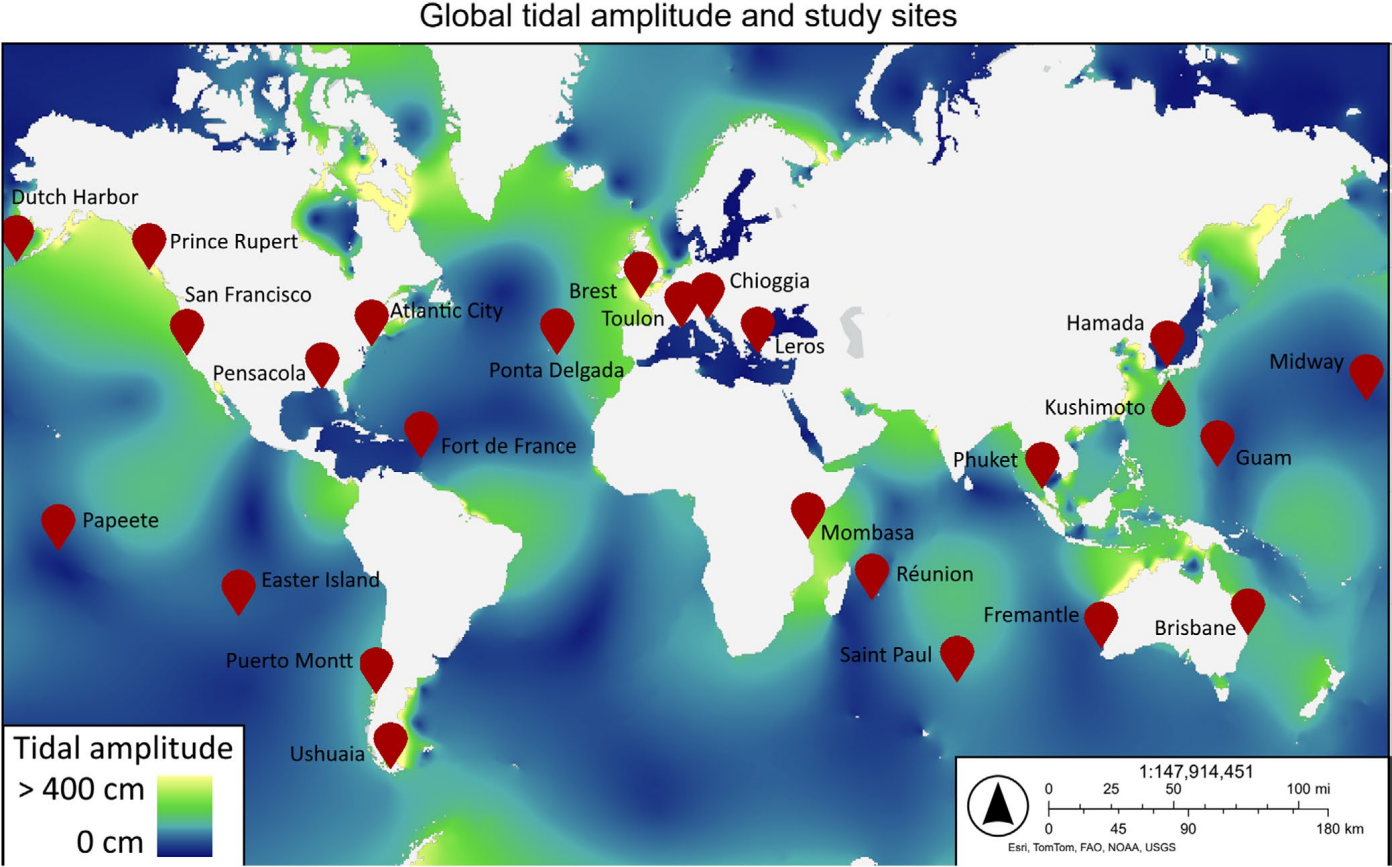
World map of tidal amplitudes calculated as 2*(M2 + S2 + K1 + O1) from FES2014 (AVISO+) along with the locations of the tidal gauge data stations used in this study. M2: Principal lunar‐semidiurnal constituent, S2: Principal solar‐semidiurnal constituent, K1: Lunisolar‐diurnal constituent, O1: Lunar‐diurnal constituent (Devlin et al. 2017; Doodson 1921).

We collected sea level data from 25 locations worldwide (Figure 1), representing a range of tidal regimes across both open ocean and semi‐enclosed seas. For 22 of these locations, we extracted high frequency (h^−1^) sea level data from the University of Hawai’i Sea Level Center and SHOM's REFMAR network (Pointe des Galets, Réunion; Caldwell et al. 2015; SHOM ‐ REFMAR 2015). These datasets span from the beginning of 1993 to the end of 2024, providing over 30 years of historical values. In addition, we included three Mediterranean locations—Toulon, Chioggia, and Leros—where we obtained very high frequency (min^−1^) sea level data from SHOM (REFMAR), ISPRA Ambiente (National Mareographic Network), and the Hellenic Navy Hydrographic Service, respectively. These datasets extend from 1993 through December 2024 (Toulon), December 2023 (Chioggia), and June 2023 (Leors) and offer greater temporal resolution. In the semi‐enclosed, microtidal regime of the Mediterranean Sea, the limited tidal range and meteorological forcing combine to increase the likelihood of Dry Tides. Data were checked for aberrant values and sea level was converted into cm above Lowest Astronomic Tide.

**FIGURE 2:**
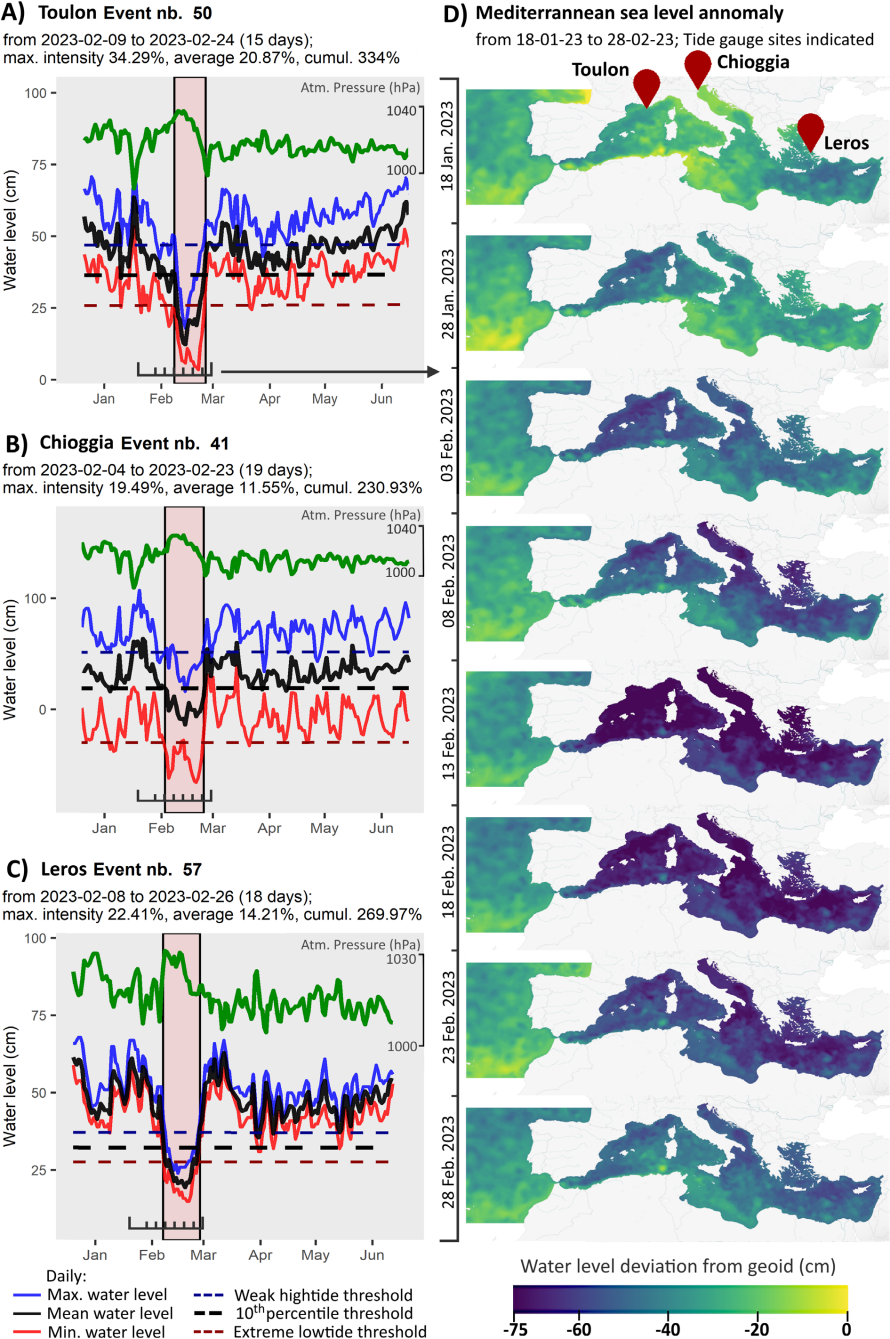
Focus on the 2023 Dry Tide event in the Mediterranean Sea. Tide gauge data (maximum daily water level, mean daily water level, minimum daily water level), relevant thresholds for nSLA/Dry Tide events, and atmospheric pressure data from December to June for: (A) Toulon; (B) Chioggia; and (C) Leros. Red boxes indicate the Dry tide event. Water level deviations from geoid based on satellite data (Clementi et al. 2021) are shown for the duration of the Dry Tide event for the Mediterranean basin (D). Map lines delineate study areas and do not necessarily depict accepted national boundaries.

Worldwide tidal amplitudes based on the Finite Element Solution 2014 tidal (FES2014; Lyard et al. 2021) model were obtained from ArcGIS online (Figure 1; AVISO+; Noveltis, Legos, CLS, Cnes). These are computed as tidal range = 2*(M2 + S2 + K1 + O1) representing the combined lunar and solar semi‐diurnal and diurnal harmonic constituents: M2 is the principal lunar‐semidiurnal constituent, S2 the principal solar‐semidiurnal constituent, K1 the lunisolar‐diurnal constituent, and O1 the lunar‐diurnal constituent (Devlin et al. 2017; Doodson 1921). For more detail on the Mediterranean locations, Mediterranean Sea Physics Analysis and Forecast data were collected from E.U. Copernicus Marine Service Information and visualized in MyOcean Pro (Clementi et al. 2021). Hourly water level anomalies (deviation from geoid) were inspected and mapped for the whole Mediterranean Sea during the 2023 Dry Tide event (Figure 2). Additionally, atmospheric pressure data were collected from the ISPRA Ambiente CNR Oceanic platform (45° 18′ 51,27” N; 12° 30′ 29,93″ E; 30 km away from Chioggia) for the entire length of the records (2007–2023), from the NOANN network of the National Observatory of Athens from Samos (37°45′21,21” N, 026°58′13,02″ E; 70 km away from Leros; Lagouvardos et al. 2017) for the entire length of the records (2010–2024), and from the SYNOP—OMM—MeteoFrance Données Essentielles dataset, Marignane weather station (43°26′15.60”N, 005°12′57.58″ E; 70 km away from Toulon) during 2010–2024. These weather stations, with accessible long‐term data, are the closest (< 70 km) to the tidal gauge stations at Chioggia, Leors, and Toulon. Given that meteorological phenomena typically occur at scales much larger than the distances between these stations and the tidal gauges, we assume the atmospheric pressure measurements adequately represent local conditions.

### 2.3 Analysis of Tidal Data

The analysis of tidal datasets was performed in the R software (ver. 4.1.3; R Core Team 2020) and the script is provided in the Supporting Information. Plots were obtained via ‘*ggplot2*’ (Wickham 2016). The average daily water level was calculated, and the highest and lowest measured tide levels (peak high and peak low tides) were identified for each day based on actual observations rather than theoretical predictions. For each of these three variables, a linear model was fitted as a function of date to account for sea level rise. To capture the effect of the lunar nodal cycle on average daily water levels as well as peak tides, a 18.6‐year sinusoid was superimposed on the linear model on all three parameters. The 18.6‐year lunar nodal cycle effect is stronger for diurnal constituents such as K_1_ and O_1_ (10%–20%) and weaker for the dominant semidiurnal tide M_2_ and associated constituents (2%–4%; Pineau‐Guillou et al. 2021), effectively resulting in a tidal modulation limited to a few percent of tidal amplitude (Pineau‐Guillou et al. 2021; Shaw and Tsimplis 2010). This modulation affects tidal range rather than average sea level and should thus have limited impact on the occurrence of transient water level depressions since higher high tides and lower low tides compensate each other in average. However, a significant multidecadal 18.6 year. signal can nevertheless appear in daily average, daily maximum, or daily minimum water levels due to the misalignment of diurnal tidal asymmetry and the calendar day, warranting the inclusion of this cycle in the models. The 10th percentile of the model's lowest residuals were subtracted from the predicted values (representing an evolving average) to obtain a linear function describing the evolving percentile thresholds for each variable. This helps to account for sea‐level rise and the lunar nodal cycle, which could otherwise obscure the detection of dry tides over time. Tidal amplitudes were determined from the difference between the 99th and 1st percentile of residuals of daily highest and lowest water levels to exclude extreme values that are not part of the astronomical tidal cycle (floods, tsunamis, aberrant values). Our script then automatically scanned for periods during which the average daily sea level was below its 10th percentile threshold, and either the daily maximum or minimum water level was also below its respective threshold.

We included nSLA events in the summary table to facilitate exploration by interested readers; however, all subsequent analyses focused only on Dry Tides (i.e., ≥ 5 days) to maintain clarity and conciseness. Each identified event was compiled into a summary table, including the following information: (1) start date; (2) end date; (3) duration; (4) event type (nSLA or Dry Tide); (5) maximum intensity (maximum difference between the average water level and its threshold for a given date); (6) average intensity over the duration of the event; (7) cumulated intensity (sum of the daily differences between the average water level and its 10th percentile threshold, calculated for each day of the event). Because the impact of a Dry Tide is relative to the local tidal regime (e.g., 20 cm is substantial in a 40 cm regime but minor in a 400 cm one) we expressed event intensity as a percentage of the maximum tidal amplitude at each location. The script automatically plotted each event for data exploration. At this stage, events caused by aberrant data or material errors (e.g., Millenium bug on the Brest tidal gauge; see Figure S1) were filtered out and the tables/plots rearranged. The table was then summarized with top (highest of all time), average, and SE for each relevant metric at each location (Table 1).

**TABLE 1:** Synthesis table on Dry Tides at the 25 study locations (see also Figure 1). Tidal amplitude expressed as cm. or Asterisk * in tidal amplitude indicate semi‐enclosed seas. Frequency expressed as events per year. Other Dry Tide characteristics expressed as: Maximal value (mean value ± standard error). Significant increase or decrease in time (linear model, *p*‐values manually adjusted to account for multiplicity, absent because none are significant).

Location characteristics			Dry Tide characteristics				
Location	Coordinates	Tidal amp.	Frequency	Duration (days)	Max. intensity (% of max. tidal amp.)	Av. intensity (% of max. tidal amp.)	Cum. intensity (% of max. tidal amp.)
Chioggia	45° 13′ 25.0” N	168*	1.3 (41 in 31 years)	24 (10.5 ± 4.9)	19.5 (9.5 ± 3.9)	11.5 (5.3 ± 2.2)	230.9 (65.6 ± 52.5)
Italy (Adriatic Sea)	12° 16′ 51.9″ E						
Toulon	43° 7′ 2.0” N	71*	1.5 (48 in 31.9 years)	40 (11.6 ± 7)	34.3 (16.1 ± 5.6)	20.9 (8.6 ± 2.9)	435.7 (111.4 ± 83.4)
France (Mediterranean)	5° 54′ 47.1″ E						
Leros	37° 07′ 50.8” N	57*	1.9 (57 in 30.4 years)	35 (10.2 ± 5.7)	37 (19.5 ± 7.5)	22.7 (10.8 ± 4.1)	544.9 (127.7 ± 102.4)
Greece (Aegean Sea)	26° 50′ 54.7″ E						
Brest	48° 22′ 58.8” N	725	0 (0 in 32 years)	0	0	0	0
France (West coast)	4° 30′ 0.0” W						
Ponta Delgada	37° 44′ 6.0” N	187	0.4 (12 in 29 years)	43 (12.2 ± 11)	9.5 (6 ± 1.6)	5.3 (3.7 ± 0.9)	149.5 (44.6 ± 37.2)
Portugal (Acores)	25° 40′ 22.8” W						
Fort de France	14° 35′ 60.0” N	64*	1.8 (34 in 19 years)	44 (11.4 ± 8.5)	24.4 (11.4 ± 5.2)	13.5 (5.9 ± 2.9)	309.1 (74 ± 64.9)
France (Caribbean)	61° 4′ 1.2” W						
Pensacola	30° 24′ 10.8” N	113*	1 (33 in 32 years)	17 (7.3 ± 2.9)	29.2 (18.7 ± 6)	18.8 (9.8 ± 3.7)	180.6 (78.2 ± 39.5)
USA (Gulf of Mexico)	87° 12′ 46.8” W						
Atlantic City	39° 21′ 18.0” N	253	0.6 (20 in 32 years)	16 (7.2 ± 2.8)	29.5 (13 ± 6.2)	11.5 (6.4 ± 2.5)	127.9 (51.7 ± 30.4)
USA (East Coast)	74° 25′ 4.8” W						
Mombasa	4° 4′ 12.0” S	397	0 (0 in 32 years)	0	0	0	0
Kenya	39° 39′ 25.2″ E						
Réunion	20° 56′ 06” S	100	1.1 (17 in 15 years)	40 (15.2 ± 11)	14.1 (7.6 ± 3)	7.2 (4.4 ± 1.5)	275 (80.2 ± 76.9)
France (Indian Ocean)	55°17′06″ E						
Fremantle	32°2′60″ S	136	1.3 (43 in 32 years)	11 (6.3 ± 1.7)	20.1 (11.3 ± 4.3)	11.6 (6.4 ± 2.4)	93.4 (46.8 ± 21.2)
Australia (West Coast)	115° 43′ 58.8″ E						
Brisbane	27° 22′ 1.2” S	265	0.7 (22 in 32 years)	10 (6.6 ± 1.3)	9 (4.6 ± 1.5)	4.6 (2.9 ± 1)	36.9 (21.3 ± 8.2)
Australia (East Coast)	153° 10′ 1.2″ E						
Phuket	7° 49′ 55.2” N	320	0.6 (18 in 32 years)	17 (8.2 ± 3.9)	9 (4.2 ± 1.9)	6.4 (2.8 ± 1.5)	108.5 (27.1 ± 25.4)
Thailand	98°25′30″ E						
Hamada	34° 53′ 60.0” N	103*	0.8 (27 in 32 years)	26 (9.6 ± 5.9)	34.8 (15 ± 6.2)	11.9 (6.8 ± 2.1)	190.9 (66.7 ± 42.5)
Japan (West Coast)	132° 4′ 1.2″ E						
Kushimoto	33° 28′ 1.2” N	226	0.8 (26 in 32 years)	10 (6.3 ± 1.6)	8.6 (5.4 ± 1.5)	5.9 (3.2 ± 1)	44 (22 ± 8.1)
Japan (East Coast)	135° 46′ 58.8”						

Location characteristics			Dry Tide characteristics				
Location	Coordinates	Tidal amp.	Frequency	Duration (days)	Max. intensity (% of max. tidal amp.)	Av. intensity (% of max. tidal amp.)	Cum. intensity (% of max. tidal amp.)
Guam	13° 25′ 58.8” N	130	1.3 (43 in 32 years)	77 (18 ± 17.2)	22.7 (7 ± 4.4)	11.6 (4.2 ± 2.6)	595.9 (101.7 ± 134.4)
USA (Pacific)	144°39′0″ E						
Midway	28° 13′ 1.2” N	88	1.6 (50 in 32 years)	73 (12.3 ± 11.8)	15.4 (7.2 ± 3)	8.8 (4 ± 1.7)	648.1 (61.1 ± 94)
USA (Pacific)	177° 22′ 1.2” W						
Papeete	17° 31′ 30” S	52	1.5 (47 in 31.2 years)	59 (13.9 ± 10.4)	23.1 (9.9 ± 4.9)	12.6 (5.4 ± 2.6)	553.9 (95.4 ± 115.5)
French Polynesia	149° 34′ 1.2” W						
Easter Island	27°8′60″ S	106	1.2 (39 in 32 years)	97 (13.2 ± 17.4)	18.6 (7.1 ± 3.5)	11.6 (4 ± 2.1)	940.7 (73 ± 160.4)
Chile	109° 26′ 52.8” W						
Puerto Montt	41° 28′ 58.8” S	705	0 (1 in 31.3 years)	6	2.3	1.6	11
Chile	72° 58′ 1.2” W						
Ushuaia	54° 48′ 18.0” S	258	0.9 (26 in 29 years)	16 (6.8 ± 2.5)	13.9 (7.8 ± 3.1)	9 (4.7 ± 1.8)	99.6 (36 ± 19.8)
Argentina	68° 17′ 42.0” W						
San Francisco	37° 48′ 25.2” N	273	0.8 (24 in 32 years)	14 (6.2 ± 2)	6.2 (3.6 ± 1.4)	3.8 (2 ± 0.8)	36.3 (14.2 ± 7.4)
USA (West Coast)	122° 27′ 54.0” W						
Prince Rupert	28° 13′ 1.2” N	731	0 (0 in 32 years)	0	0	0	0
Canada (West Coast)	177° 22′ 1.2” W						
Dutch Harbor	53° 52′ 48′ ‘N	216	0.9 (28 in 32 years)	16 (7.4 ± 2.7)	14.4 (8 ± 3)	9.3 (4.7 ± 2)	65.6 (37.7 ± 16.1)
USA (Bering Sea)	166° 32′ 16.8” W						
Saint Paul	38° 42′ 43.2” S	196	0.8 (22 in 27.3 years)	16 (8.4 ± 3.4)	12.7 (8 ± 2.2)	7.6 (4.7 ± 1.2)	129.4 (42.6 ± 24.7)
France (Southern Ocean)	77° 32′ 16.8″ E						

### 2.4 Statistical Analysis

Sea level anomalies in coastal systems may reflect the combined effects of atmospheric pressure, wind stress, and hydrological inputs. While our tide‐gauge‐based approach does not explicitly partition these drivers, the Dry Tides identified here can be interpreted as compound low‐water disturbances. We here use atmospheric pressure as a measurable proxy for persistent anticyclonic regimes under which multiple low‐water drivers tend to co‐occur. This leads to three testable predictions:

1. Tidal amplitude should negatively influence the occurrence of Dry Tides, as stronger astronomical tides may offset or mask meteorologically driven anomalies, reducing their relative impact. To test this hypothesis, we used global data from 25 locations (treating each location as a replicate; values from Table 1) and applied linear models to assess the relationship between tidal amplitude and nine Dry Tide metrics: frequency, longest duration, mean duration, highest maximum intensity, mean maximum intensity, highest average intensity, mean average intensity, highest cumulative intensity and mean cumulative intensity (Table 2). Note the distinction between ‘mean’ and ‘average’ where mean is calculated for each location and average for each event. Because multiple comparisons were involved, *p*‐values were adjusted using the Benjaminji‐Hochberg correction to control for the false discovery rate.
2. in regions where Dry Tides occur, sea level should be negatively correlated with atmospheric pressure, reflecting a classic inverse barometer effect. To test this hypothesis, we focused on the three microtidal Mediterranean locations where Dry Tides occurred most frequently, consistent with the prediction of hypothesis #1. Using local atmospheric pressure data, we applied linear regression models to assess the effects of atmospheric pressure, location, and their interaction on sea level across the whole length of records. We inspected the distribution of the data via the ‘*fitdistrplus’* R package (ver. 1.2–2; Delignette‐Muller and Dutang 2015). A Cullen & Frey graph based on 500 bootstrap replicates indicated slight skewness (−0.3) and kurtosis (3.5). However, the data still best fitted a normal distribution, and we chose to proceed with linear models, as they are generally robust to mild deviations from normality (Zuur et al. 2010). The model was thus retained, summarized in an ANOVA table, and plotted via an interaction plot from the ‘*interactions’* package (ver. 1.2.0; Bauer et al. 2005) with location as moderator and 95% confidence interval. The interactions were probed to identify individual slopes.
3. If high pressure anomalies become more frequent and persistent under climate change (Stott 2016; Tsimplis and Josey 2001), then the prevalence of Dry Tides should also increase over time. To test this hypothesis, we applied a linear model to assess the effect of date (used as a proxy for long‐term climate change since 1993) on five Dry Tide metrics: frequency, duration, maximum intensity, mean intensity, and cumulative intensity. These tests were conducted separately for each of the 25 locations (25 locations x 5 tests), and *p*‐values were adjusted using the Benjamini‐Hochberg correction to account for multiple comparisons.

**TABLE 2:** Results of linear regressions testing for the effect of tidal amplitude (in cm) on different characteristics of dry tides globally (25 locations = replicates). *p*‐values adjusted by a Benjamini‐Hochberg correction; *p* < 0. Top designates the highest value for a given location.

lm (formula = ‘parameter’ ~ tidal amplitude)						
Parameter	Estimate	Std. error	t value		Pr(> |t|)	p.adjust
Intercept (Frequency)	1.46	0.09	15.61		< 0.001	< 0.001***
Frequency (events per year)	−0.002	0.00	−7.67	↘	< 0.001	< 0.001***
Intercept (Longest Duration)	46.46	6.51	7.13		< 0.001	< 0.001***
Longest Duration (days)	−0.08	0.02	−3.63	↘	0.001	0.001**
Intercept (M. Duration)	12.60	0.94	13.37		< 0.001	< 0.001***
M. Duration (days)	−0.02	0.00	−5.54	↘	0.01	0.01**
Intercept (High. max. intens.)	26.04	2.36	11.05		< 0.001	< 0.001***
High. max. intens. (% of max. tidal amp.)	−0.04	0.01	−5.37	↘	0.001	0.001**
Intercept (M. max. intens.)	12.68	1.19	10.61		< 0.001	< 0.001***
M. max. intens. (% of max. tidal amp.)	−0.02	0.00	−4.96	↘	0.001	0.001**
Intercept (High av. intens.)	14.48	1.26	11.46		< 0.001	< 0.001***
High. av. Intens. (% of max. tidal amp.)	−0.02	0.00	−5.54	↘	0.001	0.001**
Intercept (M. av. intens.)	6.90	0.60	11.57		< 0.001	< 0.001***
M. av. Intens. (% of max. tidal amp.)	−0.01	0.00	−5.25	↘	0.001	0.001**
Intercept (High cum. intens.)	405.93	64.03	6.34		< 0.001	< 0.001***
High. cum. intens. (% of max. tidal amp.)	−0.73	0.21	−3.53	↘	0.002	0.002**
Intercept (M. cum. intens.)	84.48	7.13	11.86		< 0.001	< 0.001***
M. cum. intens. (% of max. tidal amp.)	−0.14	0.02	−6.01	↘	< 0.001	0.001**

*Note:* Arrows indicate increases ↗ or decreases ↘ with tidal amplitude.

Abbreviations: Av, Average; Cum, Cumulated; High, Highest; intens, Intensity; M, Mean.

*** *p* < 0.001.

** *p* < 0.01.

## 3 Results

### 3.1 Dry Tides on Global and Mediterranean Scale

More than 630 Dry Tide events were identified across the 25 study locations between 1993 and 2024 (Table 1, Figure 1). This is likely a conservative estimate, as some datasets were incomplete. Dry tides occurrence per location ranged from 0 in Mombasa (Kenya), Prince Rupert (Canada West Coast), and Brest (France West Coast) to 57 in Leros (Greece), with a global average of approximately 0.91 ± 0.55 events per year. The longest recorded Dry Tides occurred at Pacific islands: Easter Island in 2007 (Sup. Fig. 2; 97d; max. intensity 18.0%; m. intensity: 9.7%; cum. intensity: 940.7%), Guam in 2015 (77d; max. intensity 12%; m. intensity: 7.7%; cum. intensity: 595.9%) and 2018 (76d; max. intensity 13.4%; m. intensity: 6.1%; cum. intensity: 459.5%), and Midway Island in 1997 (73d; max. intensity 15.4%; m. intensity: 8.8%; cum. intensity: 648.1%). These Pacific Dry Tides roughly aligned with ENSO variations, coinciding with El Niño in the West and Central Pacific (Guam, Midway) and La Niña in the East Pacific (Easter Island). The most intense Dry Tide was recorded in Leros (Aegean Sea; Jan.‐Mar. 2008) with the highest daily water level below the 10th percentile thresholds of extreme low tides and an exceptional event duration (35 d; max. intensity: 37%; m. intensity: 15.6%; cum. intensity: 544.9%). This extreme Dry Tide was also detected, though less severely, in Toulon and Chioggia (25 d; max. intensity: 24.5%; m. intensity: 12.6%; cum. intensity: 326.9%; as well as 16 d; max. intensity: 15.6%; m. intensity: 8.25%; cum. intensity: 140.2%).

The 2023 Dry Tide (Figure 2), which stimulated this study, was the strongest (19 d; max. intensity: 19.5%; m. intensity: 11.6%; cum. intensity: 230.9%) but not the longest (24 d) in Chioggia (Figure 2A) and Toulon (Figure 2B; 15 d; max. intensity: 34.3%; m. intensity: 20.9%; cum. intensity: 334%). Satellite data show the progression of the 2023 Dry Tide, which began in the western basin and eventually extended across the entire Mediterranean basin (Figure 2D).

### 3.2 Dry Tides Correlate With Tidal Amplitude and Atmospheric Pressure

Tidal amplitudes had a strong and significant negative correlation with all Dry Tide characteristics like frequency, longest duration, average duration, etc. (Table 2; Linear Models; t‐value < −3.53; p.adj < 0.005), indicating that lower tidal ranges were associated with more intense and/or longer‐lasting Dry Tide events.

**FIGURE 3:**
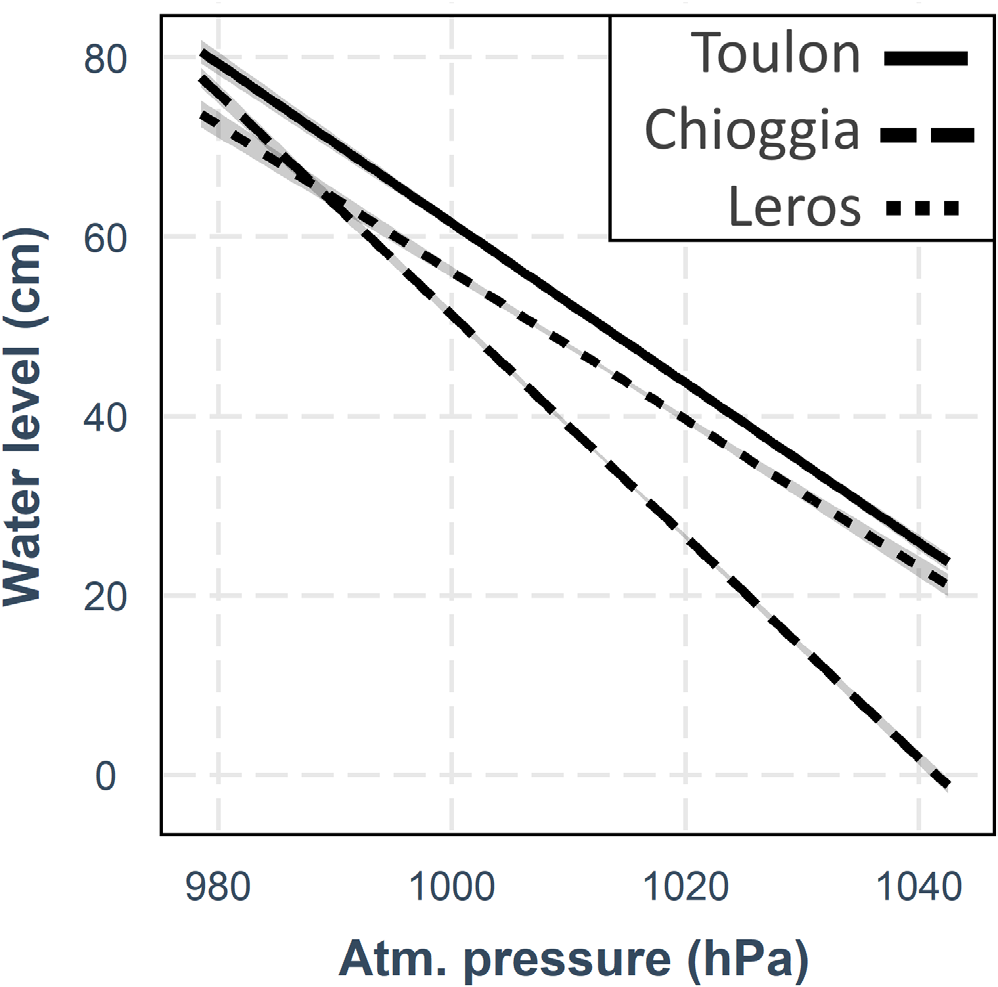
Linear models showing the correlation of Atmospheric pressure with local water levels at each location. For more details see Table 3. The 95% confidence intervals are shown as light gray, though they are barely visible since due to their narrow range.

Atmospheric pressure had a variable effect on sea level across the different locations (Linear model, Table 3), but was consistently negatively correlated with local sea level at all three microtidal Mediterranean locations, consistent with the inverse barometer effect. This relationship was confirmed by a Simple Slopes Analysis, which revealed highly significant negative effects (t‐value < −37.9; *p* < 0.001; Table 3; Figure 3).

No significant change over time (used here as a proxy of climate change) was detected for any Dry Tide characteristic at any location (Table 1).

**TABLE 3:** ANOVA table of the linear regression testing for the effect of atmospheric pressure, location, and their interaction on local mean daily water levels of the Mediterranean locations (Toulon, Chioggia, Leros). Simple slope analysis results clarify the significant interactions.

ANOVA table of linear model					
lm (formula = water level ~ location* atmospheric pressure)					
Coefficients	Df	Sum Sq.	Mean Sq.	F value	Pr(> F)
Location	1	787,377	787,377	10,402	< 0.001***
Atmospheric Pressure (hPa)	2	668,777	334,388	4418	< 0.001***
Atmospheric Pressure (hPa) : Location	2	25,751	12,875	170	< 0.001***
Residual standard error: 6.97 on 15,040 degrees of freedom					
Multiple R‐squared: 0.036, Adjusted R‐squared: 0.036					
F‐statistic: 113.7 on 5 and 15,040 DF, *p*‐value: < 2.2e‐16					
Simple Slopes Analysis					
*When* Location *= Toulon*	Estimate	Std. Error	t value	p.adj	
Conditional intercept	950.61	17.88	53.16	< 0.001***	
Slope of Atmospheric Pressure (hPa)	−0.89	0.02	−50.52	< 0.001***	
*When* Location *= Chioggia*					
Conditional intercept	1284.81	15.29	84.04	< 0.001***	
Slope of Atmospheric Pressure (hPa)	−1.23	0.02	−81.92	< 0.001***	
*When* Location *= Leros*					
Conditional intercept	876.74	21.95	39.95	< 0.001***	
Slope of Atmospheric Pressure (hPa)	−0.82	0.02	−37.89	< 0.001***	

*** *p* < 0.001.

* *p* < 0.05.

## 4 Discussion

### 4.1 Dry Tides—A Common, Globally Occurring but Overlooked Disturbance

Despite their potential for ecological disruption, Dry Tides, prolonged periods of anomalously low sea levels, have received little scientific attention, remaining undefined and largely unmonitored until now. This study provides the first quantitative definition and assessment of Dry Tides on a global scale. By analyzing more than 30 years of sea level data from 25 coastal locations, we reveal that Dry Tides are not rare anomalies, but rather recurrent events in many regions, particularly in microtidal and semi‐enclosed seas. In most locations, Dry Tides occurred nearly once per year, with some locations, such as Leros (Greece), experiencing up to two events annually, while others did not experience any during the last 30 years (i.e., Mombasa, Kenya). This frequency is comparable to that of marine heatwaves (Hobday et al. 2016), validating the chosen thresholds from a statistical point of view (too many or too few detections could indicate too liberal or conservative thresholds).

The definition developed here allowed us to detect both moderate and extreme events, including particularly extreme events in which even daily high tides fell below the threshold of extreme low tides. These were particularly common in certain locations (i.e., Leros, Greece), suggesting site‐specific vulnerability. A formalized definition can now support future research into such effects and improve the capacity for event detection and monitoring across systems.

### 4.2 Meteorological and Compound Drivers of Dry Tides

Transient weather patterns alter sea levels on short time scales, as winds, precipitations, and atmospheric pressure cause local anomalies (Hoarau et al. 2023; Weisse et al. 2021; Zamir et al. 2018). Storm surges can generate meter‐high peaks and depressions of water levels (So et al. 2019), while other extreme weather events, such as heat waves, can cause more subtle sea level variations (Mohamed et al. 2019). Our analysis revealed that the emergence of Dry Tides depends on the balance between transient atmospheric forcing and the strength of local astronomical tides.

The frequency, duration, and intensity of Dry Tides all declined significantly with increasing tidal amplitude. In macrotidal regimes (≥ 400 cm amplitude), such as Brest (France), Mombasa (Kenya), Prince Rupert (Canada West Coast), and Puerto Montt (Chile), Dry Tides were rarely detectable, likely because strong astronomical tidal forcing masks meteorological anomalies.

In contrast, in microtidal seas, such as the Mediterranean Sea, sea level variability is strongly associated with persistent atmospheric pressure anomalies (e.g., the North Atlantic Oscillation Tsimplis and Josey 2001), explaining why in these areas Dry Tides were particularly frequent. Our analysis of the Mediterranean locations revealed a strong negative correlation between atmospheric pressure and water level, indicating that Dry Tides tend to occur during periods of sustained high atmospheric pressure. Rather than implying a single causal mechanism, these results support the interpretation of Dry Tides as compound low‐water disturbances in which atmospheric forcing plays a dominant and measurable role. Persistent high‐pressure regimes are often associated with weak winds and reduced precipitation, which may coincide with decreased river discharge, thereby reinforcing local water‐level depressions. A notable example is the February 2023 Dry Tide event, the strongest intensity event on record in Toulon and Chioggia. This event coincided with a strong and persistent anticyclonic system moving from NW Europe to the Black Sea/Aegean Sea. The high‐pressure anomalies recorded at nearby weather stations closely tracked the observed drops in sea level. The large spatial extent of this anticyclonic system also explains the basin‐wide water level depression across the Mediterranean, highlighting how large‐scale atmospheric regimes can generate widespread compound low‐water disturbances.

The association between Dry Tides and persistent atmospheric regimes suggests that such events may be anticipated using standard weather forecasts. Atmospheric pressure patterns, especially sustained cyclonic and anticyclonic systems, are routinely monitored and predicted with increasing accuracy, implying that the likelihood of occurrence of Dry Tides could be forecasted several days in advance. At a local scale, additional information on river discharge and hydrological conditions could further refine such predictions, especially in semi‐enclosed or lagoonal environments. This opens the door to early‐warning systems that could help manage the ecological and operational impacts of sudden low sea level events, particularly in sensitive coastal and marine ecosystems, or inform proactive management actions, such as identifying favorable periods for vegetation establishment and restoration. Incorporating Dry Tide risk assessments into marine forecasting models could therefore provide valuable support for fisheries, aquaculture, maritime transport, and conservation efforts in regions where such events are recurrent.

### 4.3 Relationships Between Dry Tides and Climate Change

Our analysis found no consistent evidence of increasing frequency, duration, intensity, or any other Dry Tide metrics across the 25 study locations. This result was unexpected, given that other climatic disturbances, such as marine heatwaves, have notably increased over the past three decades (Frölicher et al. 2018; Smith et al. 2023). Further, previous studies have reported an increase in the frequency of weather conditions considered favorable to Dry Tides over similar timescales (Zamir et al. 2018), especially in the Mediterranean, where we observed a strong negative correlation between atmospheric pressure and sea level. While Dry Tides are clearly associated with persistent high pressure systems, the absence of a detectable climate‐driven increase in their occurrence suggests that their expression is modulated by additional environmental and oceanographic factors, including interactions among atmospheric forcing, tidal dynamics, and hydrological conditions.

Wind stress, storm‐track shifts, seasonal pressure variability, precipitations and river runoff, or changes in the vertical structure of the atmosphere may interact in complex ways to influence Dry Tide development. Local bathymetry and coastal morphology may also buffer or amplify sea level responses to atmospheric forcing, further complicating the detection of long‐term trends. These findings highlight the importance of considering both large‐scale atmospheric patterns and site‐specific physical characteristics when assessing future Dry Tide behaviour under climate change.

An important additional consideration is the interaction between Dry Tides and ongoing sea‐level rise. Although many intertidal organisms can adjust their vertical distribution with long‐term sea level variation (Kaplanis et al. 2024), rising mean sea level may, in some settings, reduce the frequency of aerial exposure for present‐day intertidal habitats, potentially dampening the ecological expression of moderate Dry Tide events. However, this effect is unlikely to universally mask Dry Tide impacts. In many coastal systems, especially where shoreward habitat migration is constrained by coastal infrastructure or steep topography, sea level rise leads to habitat compression (coastal squeeze; Pontee 2013) and increased ecological sensitivity to extremes. Under such conditions, even transient or moderate low‐water anomalies may continue to induce stress by exposing organisms adapted to higher baseline immersion regimes.

Moreover, sea‐level rise does not preclude the occurrence of extreme negative sea‐level anomalies relative to the contemporaneous mean. Because Dry Tides are defined relative to evolving local baselines, they remain ecologically relevant even under rising mean sea level. Rather than eliminating Dry Tide impacts, sea‐level rise is therefore likely to alter the ecological contexts in which they operate. Understanding how Dry Tides interact with sea‐level rise and coastal squeeze will be critical for predicting future vulnerability of intertidal and shallow subtidal ecosystems.

### 4.4 Biological and Ecological Impacts of Dry Tides

Although Dry Tides, as defined here, have not yet been systematically examined as a distinct disturbance category, a substantial body of literature documents ecological responses to nSLA across a range of coastal habitats. These studies provide a basis for inferring the types of biological responses likely to arise during Dry Tide events.

In soft‐sediment systems, temporary depressions in water level can modify sediment stability and exposure duration, thereby influencing plant establishment and community trajectories. Experimental and observational studies have shown that short periods of reduced inundation can facilitate seed germination and vegetation colonization, potentially driving transitions from unvegetated mudflats to salt marsh or mangrove systems (Balke et al. 2011, 2014; Hu et al. 2015). Conversely, extended or repeated low‐water conditions may also induce physiological stress in established wetland vegetation, including desiccation‐driven mangrove dieback (Duke et al. 2022). Such habitat transitions can alter associated invertebrate communities, for example by reducing suitable habitat for mudflat specialists following increases in surface elevation and vegetation cover (Mazik et al. 2010). These examples illustrate that prolonged low‐water conditions can reorganize benthic communities and ecosystem states, with outcomes that depend on local geomorphology, hydrology, and biological traits.

In coral reef systems, extreme and prolonged low tides have long been recognized as a major source of physical and physiological stress. Episodes of aerial exposure during exceptionally low tides have caused mass mortality of corals and other reef organisms (Anthony and Kerswell 2007; Fadlallah et al. 1995; Glynn 1968; Raymundo et al. 2017), particularly when coinciding with high irradiance, elevated air and water temperatures, or cold‐weather anomalies (Anthony and Kerswell 2007; Fadlallah et al. 1995; Hoegh‐Guldberg et al. 2005; Raymundo et al. 2017). Even without direct emersion, depressed water levels can increase light stress, reduce water exchange, elevate temperature, and lower oxygen concentrations, further intensifying physiological stress (Glynn 1968; Yamaguchi 1975). Prolonged sequences of such conditions have been reported during El Niño events in the Pacific (Ampou et al. 2017; Yamaguchi 1975), in the Indian Ocean (Brown et al. 2002; Buckee et al. 2020; Hoarau et al. 2023), and under persistent anticyclonic regimes in the Red Sea (Fishelson 1973; Loya 1972, 1976), where they resulted in substantial mortality of reef‐building corals. For example, successive Dry Tides totaling 67 days at Réunion Island were associated with a~50% reduction in coral cover (Hoarau et al. 2023), while a Dry Tide in Indonesia in 2016 led to mortality exceeding 80% on reef flats (Ampou et al. 2017).

Comparable stress mechanisms are likely to operate in other intertidal and shallow subtidal habitats. Although direct evidence remains limited, seagrasses are known to be sensitive to thermal stress, high irradiance, and aerial exposure (Diaz‐Almela et al. 2007; Unsworth et al. 2012), suggesting potential vulnerability to prolonged low‐water events. On rocky shores, species distributions are strongly structured along vertical gradients of emersion tolerance (Audouin and Milne‐Edwards 1832; Peres and Picard 1964; Rilov and Schiel 2006), and experimental manipulations have demonstrated that changes in immersion duration can lead to elevated mortality (Foster 1972). Macroalgae can lose a large proportion of their water content during emersion, experiencing marked metabolic stress (Contreras‐Porcia et al. 2017), and field observations during low‐water anomalies have reported bleaching and mortality following short desiccation periods (Zamir et al. 2018). Similarly, bivalves exhibit finite tolerance to emersion, with survival strongly dependent on temperature and humidity conditions (Delorme et al. 2021; Guareschi and Wood 2020). Dry Tide events may therefore exceed physiological thresholds for a range of habitat‐forming and associated species, leading to shifts in community composition, as documented for macroalgal assemblages and grazing invertebrates during prolonged low‐water episodes (Zamir et al. 2018).

Together, these observations indicate that prolonged nSLA can act as a disturbance capable of restructuring coastal communities through a combination of direct physiological stress and indirect habitat modification. While most existing studies have addressed isolated extreme low‐tide events or specific regional anomalies, the operational definition of Dry Tides proposed here provides a framework for systematically linking such biological responses to coherent physical events. Future work explicitly integrating ecological observations with Dry Tide metrics will be necessary to quantify their frequency, severity, and ecological consequences across habitat types and biogeographic regions.

## 5 Conclusion

We identified and formalized an understudied category of disturbance, termed Dry Tides, and provided a hierarchical definition to support their consistent detection, quantification, and comparison across regions and studies. Using tide‐gauge observations from diverse coastal settings, we show that Dry Tides occur globally, but are most frequent and intense in microtidal regimes and/or semi‐enclosed seas, where meteorological forcing exerts a strong influence on sea‐level variability. Contrary to our initial expectations, we detected no consistent temporal increase in the frequency or intensity of Dry Tides over the past three decades, suggesting that their expression is complex and possibly modulated by multiple environmental factors and does not scale linearly with changes in atmospheric circulation alone.

A broad body of ecological literature demonstrates that negative sea‐level anomalies can exert strong pressures on coastal organisms and communities. Documented responses include mortality of reef‐building corals and associated fauna, shifts in macroalgal assemblages, dieback of mangrove vegetation, and reorganization of soft‐sediment communities. These studies indicate that extended low‐water conditions can act as a disturbance capable of altering community structure and ecosystem state through both direct physiological stress and indirect habitat modification. However, Dry Tides as a coherent disturbance category have not yet been systematically linked to biological observations, underscoring the need for integrated physical–ecological studies.

Despite their potentially ecological significance, Dry Tides remain poorly recognized in comparison to other climate‐related disturbances. The operational framework proposed here provides a basis for coordinated monitoring, facilitates cross‐system comparisons, and enables integration of Dry Tide metrics into broader assessments of coastal vulnerability. Because persistent atmospheric regimes associated with Dry Tides are routinely monitored and increasingly predictable, these events offer opportunities for anticipatory management, including targeted ecological surveys and the identification of periods suitable for intervention or restoration planning. Understanding how Dry Tides interact with other stressors, including marine heatwaves and sea‐level rise, will be critical for anticipating future risks and responses of intertidal and shallow subtidal ecosystems under ongoing climate change.

## Author Contributions

**Robin P. M. Gauff:** conceptualization, investigation, writing – original draft, methodology, formal analysis, data curation. **Davide De Battisti:** conceptualization, investigation, writing – original draft, methodology. **Alberto Barausse:** methodology, formal analysis, data curation, writing – review and editing. **Laura Airoldi:** writing – review and editing, funding acquisition, project administration, supervision, resources, writing – original draft.

## Funding

This work was supported by the National Biodiversity Future Center, CN00000033, Italian MUR PNRR Mission 4 Comp. 2 Invest. 1.4. PON Ricerca e Innovazione 2014‐2020 19‐G‐12540‐1.

## Conflicts of Interest

The authors declare no conflicts of interest.

## Supporting information


**Data S1:** Script Analysis Results for all sites.

## Data Availability

The data used to support the findings of this study are openly available in University of Hawai’i Sea Level Center (https://uhslc.soest.hawaii.edu/network/); SHOM's REFMAR network (https://refmar.shom.fr/donnees‐refmar‐sur‐data.shom.fr/); ISPRA Ambiente (National Mareographic Network; https://www.venezia.isprambiente.it/); ArcGIS online (https://www.arcgis.com/home/item.html?id=d5354dea41b14f0689860bf4b2cf5e8a); E.U. Copernicus Marine Service Information (https://data.marine.copernicus.eu/); NOANN network of the National Observatory of Athens (https://www.noa.gr/en/research/main‐infrastructure/meteorological‐network/); SYNOP—OMM—MeteoFrance Données Essentielles dataset (https://www.data.gouv.fr/datasets/observation‐meteorologique‐historiques‐france‐synop); and upon request to the Hellenic Navy Hydrographic Service. The result tables of the analyses of all sites are available as Supporting Information.
