# Supplementary material for: When Tides Run Dry: Exploring an Overlooked Coastal Disturbance and Its Climate Connections
Source: Glob Chang Biol. 2026 Jul 5;32(7):e70976. doi: 10.1111/gcb.70976 (PMC13334260; doi:10.1111/gcb.70976)
Supplement: Supplementary file 1 — Data S1: Script Analysis Results for all sites. [file GCB-32-e70976-s001.zip › gcb70976-sup-0002-Supinfo2@1.Dry Tides Sup mat.docx]

**When tides run dry: Exploring an overlooked coastal disturbance and its climate connections**

Robin P.M. Gauff^1,*^, Davide De Battisti^1^, Alberto Barause^1,2^, Laura Airoldi^1,2^

1 Chioggia Hydrobiological Station “Umberto D’Ancona”, Department of Biology, University of Padova, Chioggia, Italy

2 NBFC, National Biodiversity Future Center, Palermo 90133, Italy

*Corresponding author: [gauff.robin@yahoo.de](mailto:gauff.robin@yahoo.de) ; +33658431366

**Abstract.** Weakening polar temperature gradients are associated with increasingly persistent weather pattern, increasing the risk of extreme environmental events. While disturbances, such as marine heat waves, have received growing attention, others, including negative sea level anomalies, remain understudied. Prolonged and extreme low-water conditions can impose strong physiological stress on coastal organisms and alter community structure, yet such events lack a consistent operational definition, limiting their detection and assessment. Here, we define and quantify extreme negative sea level anomalies, termed Dry Tides, as anomalous (below the 10^th^ percentile of historical records) and prolonged (>5 days) water level depressions that restrict submersion of intertidal and shallow subtidal organisms. Using a global analysis of tidal gauge records from 25 locations, we show that Dry Tides occur worldwide but are most frequent and intense in microtidal and semi-enclosed systems. These events typically last one to two weeks, with extreme cases up to 97 days and reaching amplitude depressions of up to 37% of the local tidal range. Contrary to expectations based on trends in other climate-related disturbances, we detect no consistent increase in Dry Tide frequency or intensity over the past three decades, which, together with their dependence on tidal regime, suggests that Dry Tides arise from interacting physical drivers rather than a single cause. We discuss the ecological relevance of Dry Tides in light of existing ecological literature, provide tools for their identification using local tide-gauge databases, and highlight priorities for integrating physical and ecological observations to better assess their impacts under ongoing climate change.

**Keywords:** Extreme Weather; Sea Level Anomaly; Intertidal; Disturbance; Desiccation; Stress


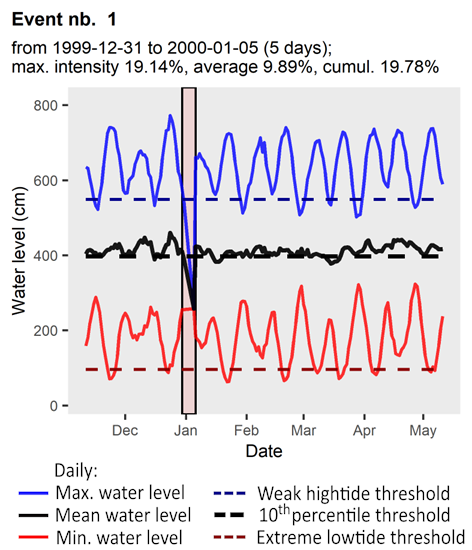


Sup. Fig. 1: Dry Tide detection caused by the millennium bug in Brest (French Atlantic). This kind of event is manually removed from the dataset before analysis.


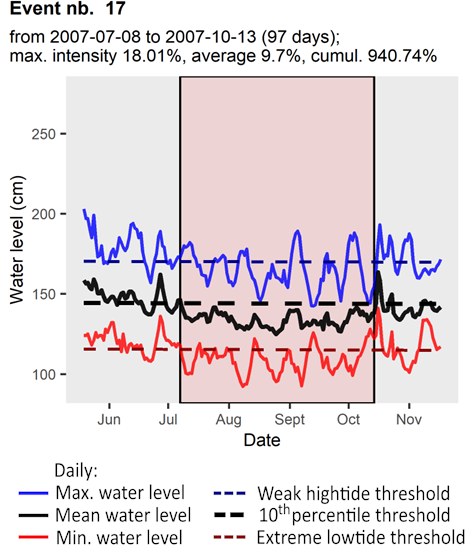


Sup. Fig. 2: Longest Dry Tide detected in our study. Location: Easter island.


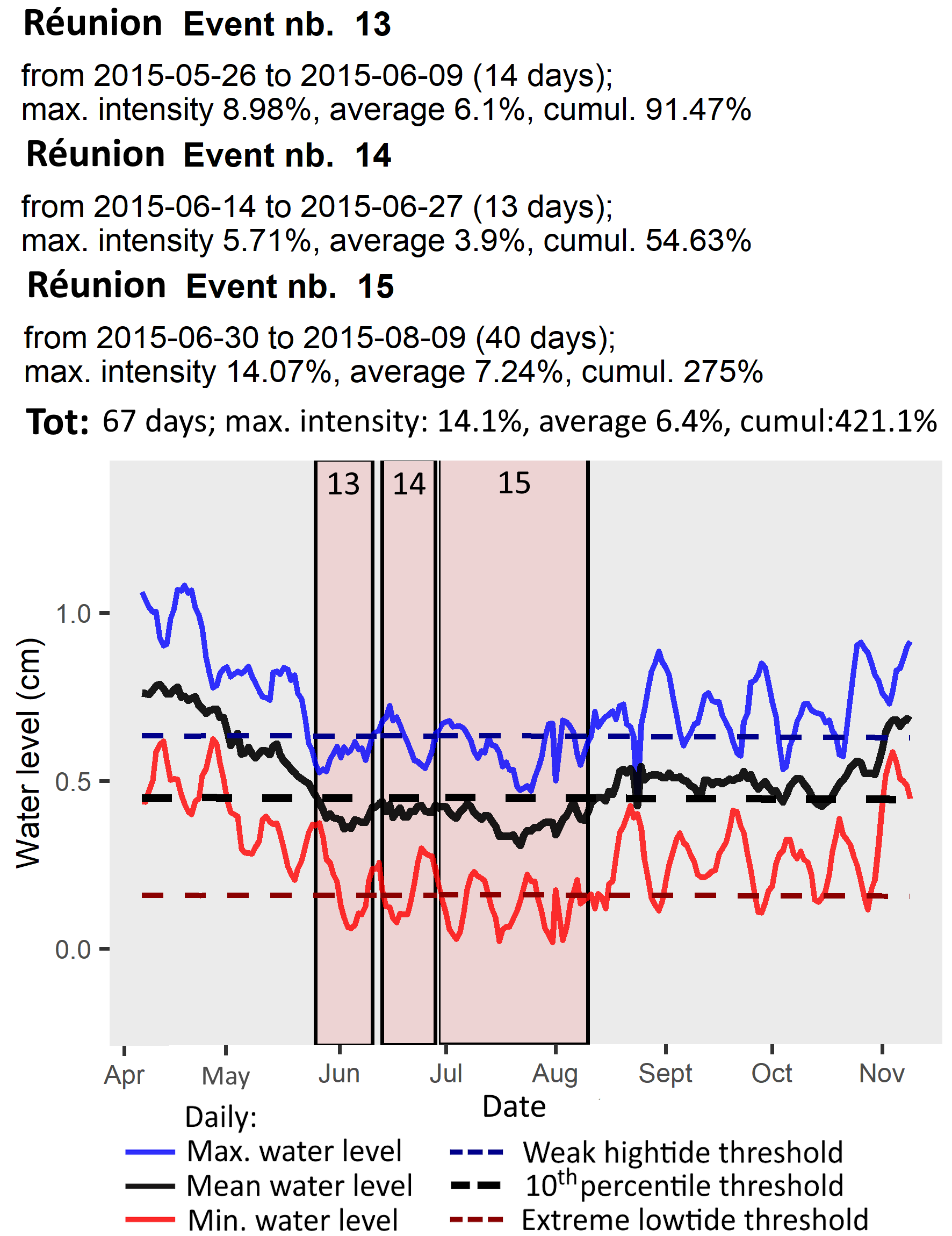


Sup. Fig. 3: Compound Dry Tide in Le Port, Réunion island. These events led to mass mortality of corals observed in Hoarau et al. (2023).
